# Simplified frontal EEG in adults under veno-arterial extracorporeal membrane oxygenation

**DOI:** 10.1186/s13613-021-00854-0

**Published:** 2021-05-13

**Authors:** Cyril Touchard, Jérôme Cartailler, Geoffroy Vellieux, Etienne de Montmollin, Pierre Jaquet, Ruben Wanono, Jean Reuter, Marylou Para, Lila Bouadma, Jean-François Timsit, Marie-Pia d’Ortho, Nathalie Kubis, Anny Rouvel Tallec, Romain Sonneville, The DINAMO Study Group, Nadine Ajzenberg, Marie‐Charlotte Bourrienne, Claire Dupuis, Dorothée Faille, Camille Legouy, Mikael Mazighi, Patrick Nataf, Katell Peoc’h, Sebastien Tanaka

**Affiliations:** 1 Department of Anesthesiology and Intensive Care, APHP, Lariboisière—Saint Louis Hospitals, 75010 Paris, France; 2 Inserm, UMRS-942, Paris Diderot University, Paris, France; 3 Université de Paris, NeuroDiderot, Inserm, 75019 Paris, France; 4 Department of Clinical Physiology, AP-HP, Bichat-Claude Bernard Hospital, 75018 Paris, France; 5 Laboratory for Vascular Translational Science, INSERM UMR1148, Team 6, Université de Paris, 75018 Paris, France; 6 Department of Intensive Care Medicine and Infectious Diseases, AP-HP, Bichat-Claude Bernard Hospital, 75018 Paris, France; 7 Department of Cardiac Surgery, Bichat-Claude Bernard Hospital, 75018 Paris, France; 8 Department of Clinical Physiology, APHP, Lariboisière - Saint Louis hospitals, DMU DREAM, 75010 Paris, France

**Keywords:** Frontal EEG, Critical care EEG, Extracorporeal membrane oxygenation, Outcome

## Abstract

**Background:**

EEG-based prognostication studies in intensive care units often rely on a standard 21-electrode montage (_std_EEG) requiring substantial human, technical, and financial resources. We here evaluate whether a simplified 4-frontal electrode montage (_4-front_EEG) can detect EEG patterns associated with poor outcomes in adult patients under veno-arterial extracorporeal membrane oxygenation (VA-ECMO).

**Methods:**

We conducted a reanalysis of EEG data from a prospective cohort on 118 adult patients under VA-ECMO, in whom EEG was performed on admission to intensive care. EEG patterns of interest included background rhythm, discontinuity, reactivity, and the Synek’s score. They were all reassessed by an intensivist on a _4-front_EEG montage, whose analysis was then compared to an expert’s interpretation made on _std_EEG recordings. The main outcome measure was the degree of correlation between _4-front_EEG and _std_EEG montages to identify EEG patterns of interest. The performance of the Synek scores calculated on _4-front_EEG and _std_EEG montage to predict outcomes (i.e., 28-day mortality and 90-day Rankin score ${\ge {4}}$) was investigated in a secondary exploratory analysis.

**Results:**

The detection of EEG patterns using _4-front_EEG was statistically similar to that of _std_EEG for background rhythm (Spearman rank test, *ρ* = 0.66, *p* < 0.001), discontinuity (Cohen’s kappa, $\kappa$ = 0.955), reactivity ($\kappa$ = 0.739) and the Synek’s score (*ρ* = 0.794, *p* < 0.001). Using the Synek classification, we found similar performances between _4-front_EEG and _std_EEG montages in predicting 28-day mortality (AUC _4-front_EEG 0.71, AUC _std_EEG 0.68) and for 90-day poor neurologic outcome (AUC _4-front_EEG 0.71, AUC _std_EEG 0.66). An exploratory analysis confirmed that the Synek scores determined by 4 or 21 electrodes were independently associated with 28-day mortality and poor 90-day functional outcome.

**Conclusion:**

In adult patients under VA-ECMO, a simplified 4-frontal electrode EEG montage interpreted by an intensivist, detected common EEG patterns associated with poor outcomes, with a performance similar to that of a standard EEG montage interpreted by expert neurophysiologists. This simplified montage could be implemented as part of a multimodal evaluation for bedside prognostication.

**Supplementary Information:**

The online version contains supplementary material available at 10.1186/s13613-021-00854-0.

## Introduction

Electroencephalography (EEG) is an almost century-old, non-invasive method deployed for electrophysiological brain investigations. In the past decade, its use has significantly increased within intensive care units (ICU) in view of improving prognostic performance after irreversible cerebral damage, secondary to a cardiac arrest [1, 2]. Recently, EEG has proven to be efficient for prognostication among septic ICU patients or those under VA-ECMO as a result of cardiogenic shock and/or refractory cardiac arrest [3, 4].

Patients under VA-ECMO experience a mortality rate close to 60% and are at risk of numerous neurological complications with dramatic consequences on the functional prognosis [5]. A recent study highlighted the role of standard 21-electrode EEG (_std_EEG) background abnormalities (slowing, discontinuity, and absence of reactivity) in such a population for early prognostication of 28-day mortality and 90-day functional outcomes [4]. Moreover, the recent use of the Synek score in other critical conditions, such as cardiac arrest [6, 7], sepsis-associated encephalopathy [3] and traumatic brain injury [8] suggests that the use of such EEG scores could be of interest in adult VA-ECMO patients.

Nevertheless, in practice, installing 21 electrodes is time-consuming and requires constant adjustments to reduce artifacts. In addition, this method requires 24/7 availability of qualified medical personnel able to install the scalp electrodes and of trained neurologists for interpretation. In this context, simplifying the EEG montage while maintaining good clinical performance at establishing prognosis would represent a considerable advantage, as it may significantly reduce the time to interpretation in clinical practice. However, whether a simplified EEG montage is associated with a more straightforward interpretation at the bedside remains unknown, as EEG interpretation (by a neurologist or an intensivist) in itself requires specific training.

A recent study confirmed that a simplified 6-electrode montage (F3, T3, P3, F4, T4 and P4) can detect major background abnormalities after cardiac arrest [9]. A simplified montage is already used to guide the depth of sedation in an ICU [10] and some studies suggest that a frontal montage could be as effective as 21 electrodes for the detection of EEG patterns associated with a poor prognosis in post-anoxic comatose patients [11]. However, the use of a simplified montage for detection of background abnormalities in adults under VA-ECMO patients has never been investigated.

We aimed to evaluate whether a simplified 4-frontal electrode montage (_4-front_EEG) could detect EEG patterns associated with poor outcomes in adult patients under VA-ECMO, as compared to interpretation made on _std_EEG recordings. Moreover, we aimed to explore the performance of Synek scores calculated on this _4-front_EEG montage to predict outcome.

## Methods

### Patients

We conducted a reanalysis of EEG data from a prospective cohort of 118 adult patients treated with VA-ECMO, in whom EEG was performed on admission to intensive care. The study was conducted in two intensive care units of the Bichat-Claude Bernard University Hospital, Paris, France, between November 2013 and November 2017 [4].

The local ethics committee (IRB 00006477, study number 14-050) approved this research. Patients meeting the inclusion criteria were cases of refractory cardiogenic shock or refractory cardiac arrest requiring at least 24 h of VA-ECMO and having undergone a 21-electrode EEG within the first 3 days of treatment. We analyzed baseline demographic data, clinically relevant data before VA-ECMO cannulation and on the day of the EEG monitoring, including doses of sedatives. ICU severity scores including Simplified Acute Physiology Score 2 (SAPS2), Sepsis-Related Organ Failure Assessment (SOFA) at ECMO cannulation and survival after veno-arterial ECMO were also recorded.

### Study protocol

**Fig. 1:**
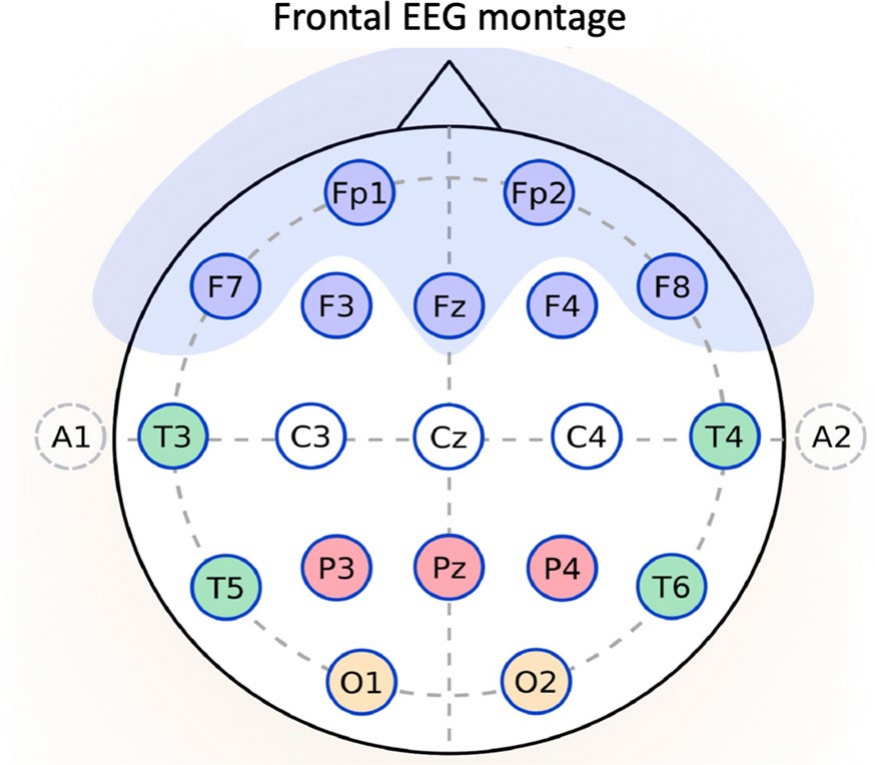
Montage description. Difference between frontal montage (4 electrodes: Fp1, Fp2, F7 and F8, blue electrodes) and standard montage (21 electrodes/system 10–20)

EEG was recorded with system plus evolution, Micromed^®^ (Modigliano Veneto, Italy). EEG recordings consisted of a 30-min standard EEG (_std_EEG) using 21 electrodes placed according to the international 10–20 system and interpreted by two neurophysiologists (RW/ART), who were blinded to the patient’s outcome. They were classified as either “reactive” or “non-reactive” as well as “continuous” or “discontinuous” and a frequency range was determined for the background rhythm, as previously described [4]. The simplified montage (_4-front_EEG) included only four electrodes from the _std_EEG montage: Fp1-Fz and F7-Fz on the left and Fp2-Fz and F8-Fz on the right (Fig. 1). Interpretation of the _4-front_EEG was performed by an intensivist (CT) who was blinded to the existing _std_EEG classification and had previously completed a 1-year training in critical care EEG interpretation.

Briefly, training consisted of regular meetings with the neurophysiologists of the Bichat Hospital (one afternoon a week), including interpretation and discussion of ICU EEG tracings. After a year of training, the analysis of the relevant 118 _4-front_EEG recordings of the study was carried out. None of the EEGs included in this study had been analyzed and/or discussed during the year of training. The intensivist evaluated on the 4-frontal electrode EEG reactivity, continuity, background rhythm and the Synek score. _4-front_EEG was compared to _std_EEG which was considered as the gold standard. In addition, we used the Synek classification to score _4-front_EEG and _std_EEG recordings between 1 (normal) and 5 (fatal) (Additional file 1) [12].

### EEG terminology

The American Clinical Neurophysiology Society’s (ACNS) standardized critical care EEG terminology was used for the study [13]. A discontinuous background activity was defined when > 10% of the record consisted of periods of lower voltage, i.e., attenuation (> 10 μV) or suppression (< 10 μV). For each patient, EEG reactivity was tested at bedside according to a standardized protocol, in line with a recent international consensus [14].

The Synek score was determined on _std_EEG and _4-front_EEG by expert neurophysiologists and by an intensivist, respectively, as described in the Additional file 1 [12].

### Outcome variables

_std_EEG, _4-front_EEG and clinical variables associated with unfavorable outcome were investigated. 28-day mortality following the start of VA-ECMO and 90-day poor functional outcome (i.e., severe disability or death at 90 days, defined by a score of 4–6 on the modified Rankin scale) were analyzed. Outcomes at 90 days were assessed during a telephone interview by a physician blinded to the EEG results [4].

### Statistical analysis

The main outcome measure was the degree of correlation between _4-front_EEG and _std_EEG montages to identify EEG patterns of interest. Values were expressed as percentages for qualitative variables and medians [inter quartile range: (IQR)] for quantitative variables. We used non-parametric Kruskal–Wallis and Mann–Whitney tests for quantitative data, and the Fisher’s test for qualitative variables. To compare interpretation of the two EEG montages, we constructed a 2 × 2 confusion matrix for reactivity and continuity, and computed Cohen’s kappa coefficients. We used dummy variables with 1 for reactivity and continuity and 0 otherwise. We then reported Fisher’s score, sensitivity and specificity. We used Spearman rank-based correlation to assess correlation between the Synek score computed from _4-front_ and _std_EEG. The significance level used in this study was *α* = 0.05.

The performance of the Synek score, calculated on _4-front_EEG and _std_EEG montages, to predict outcomes (i.e., 28-day mortality and poor 90-day functional outcome) was investigated in a secondary exploratory analysis. To evaluate propensity to predict the 28-day mortality outcome, we drew receiver operating characteristic (ROC) curves obtained from univariate logistic regressions. For each univariate regression, we provided the odds ratios (OR) with its 95% confidence interval (CI) and the areas under curves (AUC) for the ROC curves. The association between each EEG variable (background rhythm, and Synek scores on _4-front_EEG and _std_EEG) and outcomes (day-28 mortality and day-90 mRS score ≥ 4) was evaluated using logistic regression analysis after adjustment for age, non-neurological SOFA and pre-ECMO cardiac arrest, as previously described [4].

## Results

### Patients

One hundred eighteen patients [58 (48; 66) years old, 32% female] were included (Table 1). The median SOFA on admission was 12 [10; 14.75]. Thirty-five (30%) had suffered cardiac arrest before ECMO cannulation. At the time of the EEG, 106 patients were sedated (90%) and none were in therapeutic hypothermia. Sixty-two (53%) had died at 28 days (D28). The median Rankin score of the 56 patients alive at 90 days was 3 [2; 5]. Of the 21-electrode EEGs, 8% (9 patients) were unreactive, 25% were discontinuous (30 patients), the median background rhythm was 6 Hz [6, 7] and the median Synek score was 2 [2, 5]. It should be noted that no electrical seizures were detected on the 21-electrode EEGs (_std_EEG) by the experts.

**Table 1:** Patient characteristics and EEG findings with respect to 28-day survival

Variable	All patients (*n* = 118)	28-day survivors (*n* = 56)	28-day non-survivors (*n* = 62)	*p* value
At ICU admission				
Age, years	58 [48; 66]	54 [40; 61]	61 [51; 68]	< 0.001
Male gender	80 (67.8%)	36 (64.3%)	44 (71%)	0.554
BMI	25.3 [23.1; 30.2]	25.1 [23.3; 30.7]	26 [22.7; 29.7]	0.757
Pre-hospital Rankin score	1 [0; 2]	2 [0; 2]	1 [0; 2]	0.518
Charlson score	2 [0; 3]	1 [0; 2.5]	2 [1; 3]	0.075
SAPS II	57 [43; 74.5]	54 [35; 68]	60 [44.75; 78]	0.08
Pre-ECMO cardiac arrest	35 (29.7%)	15 (26.8%)	20 (32.3%)	0.55
SOFA score at ICU admission	11 [8; 13]	10 [8; 13]	11 [9; 14]	0.211
SOFA score at time of ECMO cannulation	12 [10; 15]	12 [9; 13.5]	13 [11; 15]	0.05
At time of EEG recording				
Temperature, °C	36.7 [36.2; 37]	36.8 [36.2; 37.2]	36.7 [36.2; 37]	0.359
Catecholamine infusion	107 (90.7%)	53 (94.6%)	54 (87.1%)	0.211
Neuromuscular blockade	25 (21.2%)	13 (23.2%)	12 (19.4%)	0.656
Sedation	106 (89.8%)	50 (89.3%)	56 (90.3%)	0.999
Midazolam, mg/h	5 [2; 5]	4 [0; 6]	5 [2.75; 5]	0.846
Morphine, mg/h	4 [0; 5]	4 [0; 5]	4 [0; 5]	0.474
_ std_EEG findings				
Reactivity	109 (92.4%)	54 (96.4%)	55 (88.7%)	0.167
Continuity	88 (74.6%)	49 (87.5%)	39 (62.9%)	0.002
Background rhythm (Hz)	6 [6; 7]	7 [6; 8]	6 [6; 7]	0.003
Synek score	2 [2; 5]	2 [1; 2]	2 [2; 5]	< 0.001
_4-front_EEG				
Reactivity	106 (89.8%)	53 (94.6%)	53 (85.5%)	0.131
Continuity	88 (74.6%)	50 (89.3%)	38 (61.3%)	< 0.001
Background rhythm (Hz)	7 [5; 8]	7 [6; 8]	6 [5; 7]	0.012
Synek score	2 [2; 5]	2 [1; 2]	3 [2; 5]	< 0.001

The Synek score ranges from 1 to 5, with higher scores indicating more severe encephalopathy

Data are presented as median (interquartile range) or numbers (percentages)

ICU: intensive care unit; BMI: body mass index; SAPS: Simplified Acute Physiology Score; SOFA: Sequential Organ Failure Assessment; EEG: electroencephalography; _std_EEG: 21-electrode montage; _4-front_EEG: 4-electrode montage

### Pattern concordance between _std_EEG and _4-front_EEG

**Fig.2:**
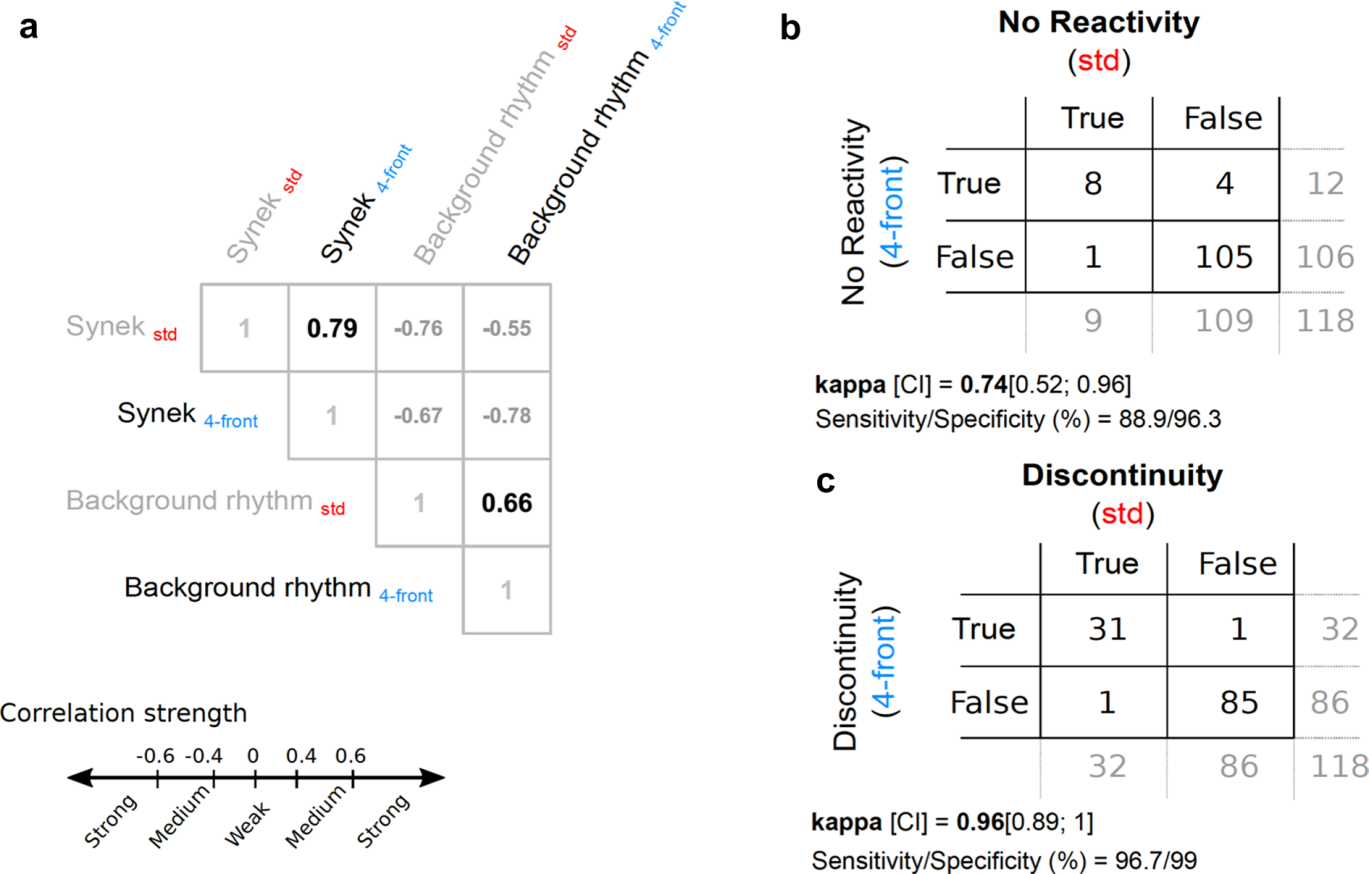
Similarity between EEG variables computed from the standard and frontal montages. **a** Spearman correlation matrix showing strong association between standard (std, red fonts) and frontal (4-front, blue fonts) derived Synek score (*ρ* = 0.79, *p* < 0.001). On the same matrix, we also show strong association between std and 4-front estimated background rhythm (*ρ* = 0.66, *p* < 0.001). Confusion tables between std and 4-front montages computed for the absence of reactivity (**b**) and the discontinuity (**c**)

Among the 118 patients, we identified 9 (7.6%) unreactive EEG patients with _std_EEG versus 12 (10.2%) with the _4-front_EEG. The accuracy of the frontal monitoring to detect reactivity compared to the standard montage was statistically similar [$\kappa$ = 0.739 (0.522; 0.957)]. We then compared how the continuity of the EEG signal could be assessed based on the _4-front_EEG montage; both frontal and standard classifications were similar [$\kappa$ = 0.955 (0.894; 1)]. Concerning the background rhythm, we reported statistically similar results, with 6 Hz [6, 7] and 7 Hz [5, 8] (*r* = 0.655, *p* < 0.001, Spearman rank test) for frontal and standard montages, respectively. Finally, we compared the Synek score estimated from _4-front_EEG and _std_EEG. Synek scores between the two groups were not significantly different (*p* non-significant, Wilcoxon signed-rank paired test) and were strongly correlated (*r* = 0.795, *p* < 0.001, Spearman rank test) (Fig. 2).

### _std_EEG vs _4-front_EEG to predict mortality and Rankin-based neurological outcome

We found that discontinuity, background rhythm and Synek scores obtained from _std_EEG and _4-front_EEG were significantly linked to 28-day mortality (Table 1, *p* < 0.005). In addition, we found that for both _std_EEG and _4-front_EEG, the Synek scores were significantly correlated with continuity (*r* = − 0.783 and *r* = − 0.762) and background rhythm (*r* = − 0.776 and *r* = − 0.782, all *p* < 0.0001, Spearman rank correlation) (Fig. 2). Therefore, we decided to retain only the Synek score as the EEG marker, which was the feature with the best-balanced performance to analyze the prediction using the ROC curve.

**Fig. 3:**
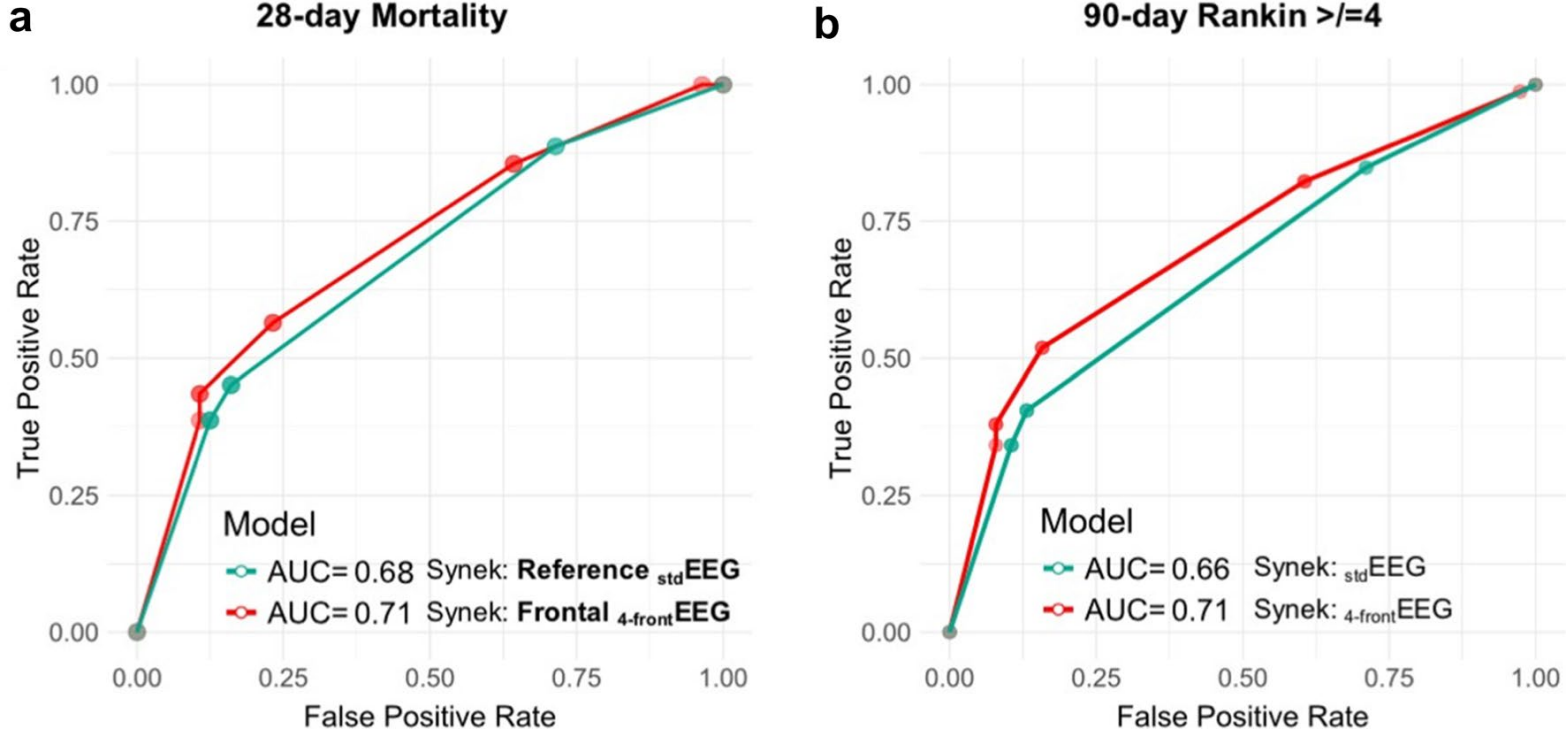
Performance of standard (_std_EEG) and frontal (_4-front_EEG) montages for prediction of 28-day mortality and 90-day poor functional outcome. The Synek score, determined by a trained intensivist on 4 frontal electrodes, was associated with 28-day mortality (AUC = 0.71, specificity 0.77 and sensitivity 0.57) and 90-day poor functional outcome (Rankin score ≥ 4) (AUC = 0.71, specificity 0.82 and sensitivity 0.40) with a precision comparable to an expert’s interpretation on 21 electrodes (AUC = 0.68 specificity 0.84 and sensitivity 0.45 and AUC = 0.66, specificity 0.85 and sensitivity 0.30, respectively)

We found that the _std_EEG Synek score was associated with 28-day mortality (Fig. 3, AUC = 0.68, accuracy: 64%, *p* < 0.001, sensitivity = 0.45 and specificity = 0.84) and with a 90-day Rankin score ≥ 4 (AUC = 0.66, accuracy: 66%, *p* = 0.004, sensitivity = 0.30 and specificity = 0.85). With _4-front_EEG, the Synek score was also associated with 28-day mortality and a 90-day Rankin ≥ 4 pts (AUC = 0.71, balanced accuracy 66%, *p* < 0.001, sensitivity = 0.57, specificity = 0.77 and AUC = 0.71, 68%, *p* < 0.001, sensitivity = 0.40, specificity = 0.82, respectively). A comparison of ROC curves revealed no difference between this biomarker when 4 or 21 electrodes are used (Delong test, *p* = NS).

### Association between EEG parameters and outcomes (Table 2)

**Table 2:** Association between EEG parameters and outcomes

Outcomes	Day-28 mortality		Day-90 mRS ≥ 4	
Variable	Adjusted OR^a^	95% CI	Adjusted OR^a^	95% CI
Synek score, _4-front_EEG (per 1-point increment)	1.67	[1.25; 2.24]	1.83	[1.25; 2.70]
Synek score, _std_EEG (per 1-point increment)	1.55	[1.16; 2.07]	1.68	[1.13; 2.49]
Background rhythm _std_EEG (per 1-Hz increment)	0.71	[0.52; 0.97]	0.73	[0.52; 1.03]

The Synek score ranges from 1 to 5, with higher scores indicating more severe encephalopathy

mRS: modified Rankin scale; OR: odds ratio; EEG: electroencephalography; _4-front_EEG: 4-electrode montage; _std_EEG: 21-electrode montage

^a^The association between each EEG variable (background rhythm, and Synek’s scores on _4-front_EEG and _std_EEG) and outcomes (day-28 mortality and day-90 mRS score ≥ 4) were evaluated using logistic regression analyses adjusted for age, pre-ECMO cardiac arrest, and non-neurological SOFA score at time of cannulation

Synek scores determined by 4 or 21 electrodes were independently associated with 28-day mortality and poor 90-day functional outcome, with no significant differences in the association estimates (Table 2). Background rhythm expressed as a continuous variable (in Hertz), was significantly, independently associated with mortality at 28 days but not with poor functional outcome, as previously described [4].

## Discussion

In this study, we evaluated how poor prognosis among ICU patients under VA-ECMO could be assessed using a 4-electrode frontal EEG. Working with EEG features such as reactivity, discontinuity, background rhythm and the Synek classification, we obtained similar results in prognostication using the simplified (_4-front_EEG) as with the classical montage (_std_EEG). In particular, we confirmed that the _4-front_EEG showed comparable precision with _std_EEG for predicting 28-day mortality, but also 90-day functional outcome based on the Rankin score. In addition, we further simplify prognostication by suggesting that an intensivist with a training in neurophysiology could interpret the simplified _4-front_EEG. At the same time, our secondary exploratory analysis found that after adjustment for age, non-neurological SOFA and pre-ECMO cardiac arrest, the 4-electrode and 21-electrode Synek scores showed similar performances in predicting mortality and poor functional outcome. In summary, a trained intensivist’s interpretation of EEGs based solely on 4 frontal electrodes can, in the context of VA-ECMO patients, provide information about patient survival and neurological prognosis. This strategy is not intended to fully replace a thorough neurological examination, but may enhance patient management, and therefore prognosis, especially as the presence of neurophysiologists or personnel able to set up a classical EEG is often lacking. In our study, experienced EEG technicians placed the electrodes, but one may expect that the use of dedicated neuromonitoring devices with 4 frontal electrodes will improve patients’ management and prognostication.

On a daily basis, the prognostication in ECMO patients remains a complex task requiring clinical examination, EEG, serum biomarker assay and neuroimaging. A multimodal neurological assessment upon ICU admission appears indispensable to assist clinicians with the level of care [15, 16]. Unfortunately, within hospital structures, we are often confronted with a decrease in the number of personnel specialized in EEG and more departments are turning towards private structures at increased costs. We have demonstrated here that prognostication based on EEG can be simplified using a 4-electrode montage that consists of electrodes applied to the forehead rather than the conventional electrodes positioned on the scalp. This technical adjustment led to a simplified interpretation of the EEG, that we have shown can be made by an intensivist who has been trained for only one year. Highly malignant patterns (suppression or burst suppression) have recently predicted poor outcome without false prognostications in comatose patients after cardiac arrest [7, 17]. According to our results, 4 frontal electrodes are sufficient to detect such cortical electrical alterations and could be used for prognostication after cardiac arrest.

Our study has certain limitations that we should point out. Firstly, an important issue concerns the detection of epileptic episodes using the simplified montage. Indeed, in our cohort no seizures were detected using the 21-electrode montage. This blind spot prevented us from evaluating the simplified montage upon such a critical feature. Besides, several studies showed limited usefulness of a reduced montage for the detection of seizures that were previously identified using the full montage [18–20]. A 21-electrode set-up should therefore be considered when there is a clinical or anamnestic suspicion of an epileptic seizure in an ICU, and expert interpretation is indispensable in such a case. Furthermore, it should be noted that our study only included patients without focal brain injury. Therefore, our results may not be extrapolatable to patients with stroke or traumatic brain injury. In our study, if an unreactive EEG was perceived by both 4 and 21 electrodes, it was not associated with mortality. The maintenance of this pattern in the ICU prognostication is therefore questionable since other EEG patterns may already contain sufficient information for reliable prognostication [15, 21]. A recent study shows that reactivity testing in itself is not sufficiently reliable for poor outcome prediction. However, for prediction of good outcome, it appears to have added value [14].

We show that 4 frontal electrodes can independently predict mortality, but the method is not optimal for this type of conclusion. Indeed, 90% of the patients were under sedation at the time of the EEG. By their inhibitory properties, sedative drugs disturb the EEG and can slow down the tracing and cause burst suppression pattern. The specificity of the prediction could therefore be increased if the method imposed the absence of sedation at the time of the EEG. In addition, it should be stressed that the sensitivity of these biomarkers on mortality remains poor [4] confirmed by our results. Recent studies underline the importance of the multimodal approach combining multiple prognostication tests, in particular to improve sensitivity [22]. Further studies are therefore necessary to evaluate the differences in prognosis among VA-ECMO patients, or more generally after cardiac arrest, between 21 and 4 electrodes in a multimodal approach (combining clinical examination, blood neuron-specific enolase, and imaging).

Although a prior study showed that ACNS terminology can be used by unexperienced reviewers [23] other studies point out the importance of experience in EEG interpretation in some situations [24]. While certain patterns can be detected by intensivists after assiduous training with experts on a limited number of electrodes, reading a higher resolution trace to detect injury or functional abnormalities undeniably requires a greater expertise. More generally, the exchange of knowledge between specialties is essential to grasp all the issues involved in the management of patients in ICU and for the prognostication challenges often associated. This study is therefore part of an effort to encourage communication and the sharing of tasks between neurophysiologists and intensivists.

## Conclusion

In adult patients under VA-ECMO, a simplified 4-frontal electrode EEG montage interpreted by an intensivist, detected common EEG patterns associated with poor outcomes, with a performance similar to that of a standard EEG montage interpreted by expert neurophysiologists. This simplified montage could be implemented as part of a multimodal evaluation for bedside prognostication. These findings deserve validation in multicenter settings.

## Supplementary Information

**Additional file 1.** Synek classification.

## Data Availability

The datasets used and/or analyzed during the current study are available from the corresponding author on reasonable request.

## References

[1] Friberg H, Cronberg T, Dünser MW, Duranteau J, Horn J, Oddo M (2015). Survey on current practices for neurological prognostication after cardiac arrest. Resuscitation.

[2] Herman ST, Abend NS, Bleck TP, Chapman KE, Drislane FW, Emerson RG (2015). Consensus statement on continuous EEG in critically ill adults and children, part I: indications. J Clin Neurophysiol.

[3] Azabou E, Magalhaes E, Braconnier A, Yahiaoui L, Moneger G, Heming N (2015). Early standard electroencephalogram abnormalities predict mortality in septic intensive care unit patients. PLoS ONE.

[4] Magalhaes E, Reuter J, Wanono R, Bouadma L, Jaquet P, Tanaka S (2020). Early EEG for prognostication under venoarterial extracorporeal membrane oxygenation. Neurocrit Care.

[5] Schmidt M, Burrell A, Roberts L, Bailey M, Sheldrake J, Rycus PT (2015). Predicting survival after ECMO for refractory cardiogenic shock: the survival after veno-arterial-ECMO (SAVE)-score. Eur Heart J.

[6] Azabou E, Fischer C, Maugiere F, Vaugier I, Annane D, Sharshar T, Lofaso F (2016). Prospective cohort study evaluating the prognostic value of simple EEG parameters in postanoxic coma. Clin EEG Neurosci.

[7] Westhall E, Rossetti AO, van Rootselaar A-F, Wesenberg Kjaer T, Horn J, Ullén S (2016). Standardized EEG interpretation accurately predicts prognosis after cardiac arrest. Neurology.

[8] Chatelle C, Rosenthal E, Bodien Y, Spencer-Salmon C, Giacino J, Eldow B (2020). EEG correlates of language function in traumatic disorders of consciousness. Neurocrit Care.

[9] Backman S, Cronberg T, Rosén I, Westhall E (2020). Reduced EEG montage has a high accuracy in the post cardiac arrest setting. Clin Neurophysiol.

[10] Schneider G, Heglmeier S, Schneider J, Tempel G, Kochs EF (2004). Patient state index (PSI) measures depth of sedation in intensive care patients. Intensive Care Med.

[11] Pati S, McClain L, Moura L, Fan Y, Westover MB (2017). Accuracy of limited-montage electroencephalography in monitoring postanoxic comatose patients. Clin EEG Neurosci.

[12] Synek VM (1988). Prognostically important EEG coma patterns in diffuse anoxic and traumatic encephalopathies in adults. J Clin Neurophysiol.

[13] Hirsch LJ, LaRoche SM, Gaspard N, Gerard E, Svoronos A, Herman ST (2013). American Clinical Neurophysiology Society’s standardized critical care EEG terminology: 2012 version. J Clin Neurophysiol.

[14] Admiraal MM, van Rootselaar A-F, Hofmeijer J, Hoedemaekers CWE, van Kaam CR, Keijzer HM (2019). Electroencephalographic reactivity as predictor of neurological outcome in postanoxic coma: a multicenter prospective cohort study. Ann Neurol.

[15] Hofmeijer J, Beernink TMJ, Bosch FH, Beishuizen A, Tjepkema-Cloostermans MC, van Putten MJAM (2015). Early EEG contributes to multimodal outcome prediction of postanoxic coma. Neurology.

[16] Oddo M, Rossetti AO (2014). Early multimodal outcome prediction after cardiac arrest in patients treated with hypothermia. Crit Care Med.

[17] Guedes B, Manita M, Rita Peralta A, Catarina Franco A, Bento L, Bentes C (2020). Prognostic significance of specific EEG patterns after cardiac arrest in a Lisbon cohort. Clin Neurophysiol Pract.

[18] Rubin MN, Jeffery OJ, Fugate JE, Britton JW, Cascino GD, Worrell GA (2014). Efficacy of a reduced electroencephalography electrode array for detection of seizures. Neurohospitalist.

[19] Kolls BJ, Husain AM (2007). Assessment of hairline EEG as a screening tool for nonconvulsive status epilepticus. Epilepsia.

[20] Young GB, Sharpe MD, Savard M, Al Thenayan E, Norton L, Davies-Schinkel C (2009). Seizure detection with a commercially available bedside EEG monitor and the subhairline montage. Neurocrit Care.

[21] Sivaraju A, Gilmore EJ, Wira CR, Stevens A, Rampal N, Moeller JJ (2015). Prognostication of post-cardiac arrest coma: early clinical and electroencephalographic predictors of outcome. Intensive Care Med.

[22] Sandroni C, D’Arrigo S, Nolan JP (2018). Prognostication after cardiac arrest. Crit Care.

[23] Gaspard N, Hirsch LJ, LaRoche SM, Hahn CD, Westover MB (2014). Critical care EEG Monitoring research consortium. Interrater agreement for critical care EEG terminology. Epilepsia.

[24] Benarous L, Gavaret M, Soda Diop M, Tobarias J, de Bourmont SDG, Allez C (2019). Sources of interrater variability and prognostic value of standardized EEG features in post-anoxic coma after resuscitated cardiac arrest. Clin Neurophysiol Pract.

