# Supplementary material for: Simplified frontal EEG in adults under veno-arterial extracorporeal membrane oxygenation
Source: Ann Intensive Care. 2021 May 13;11:76. doi: 10.1186/s13613-021-00854-0 (PMC8119573; doi:10.1186/s13613-021-00854-0)
Supplement: Supplementary file 1 — Additional file 1. Synek classification. [file 13613_2021_854_MOESM1_ESM.pptx]

## Slide 1
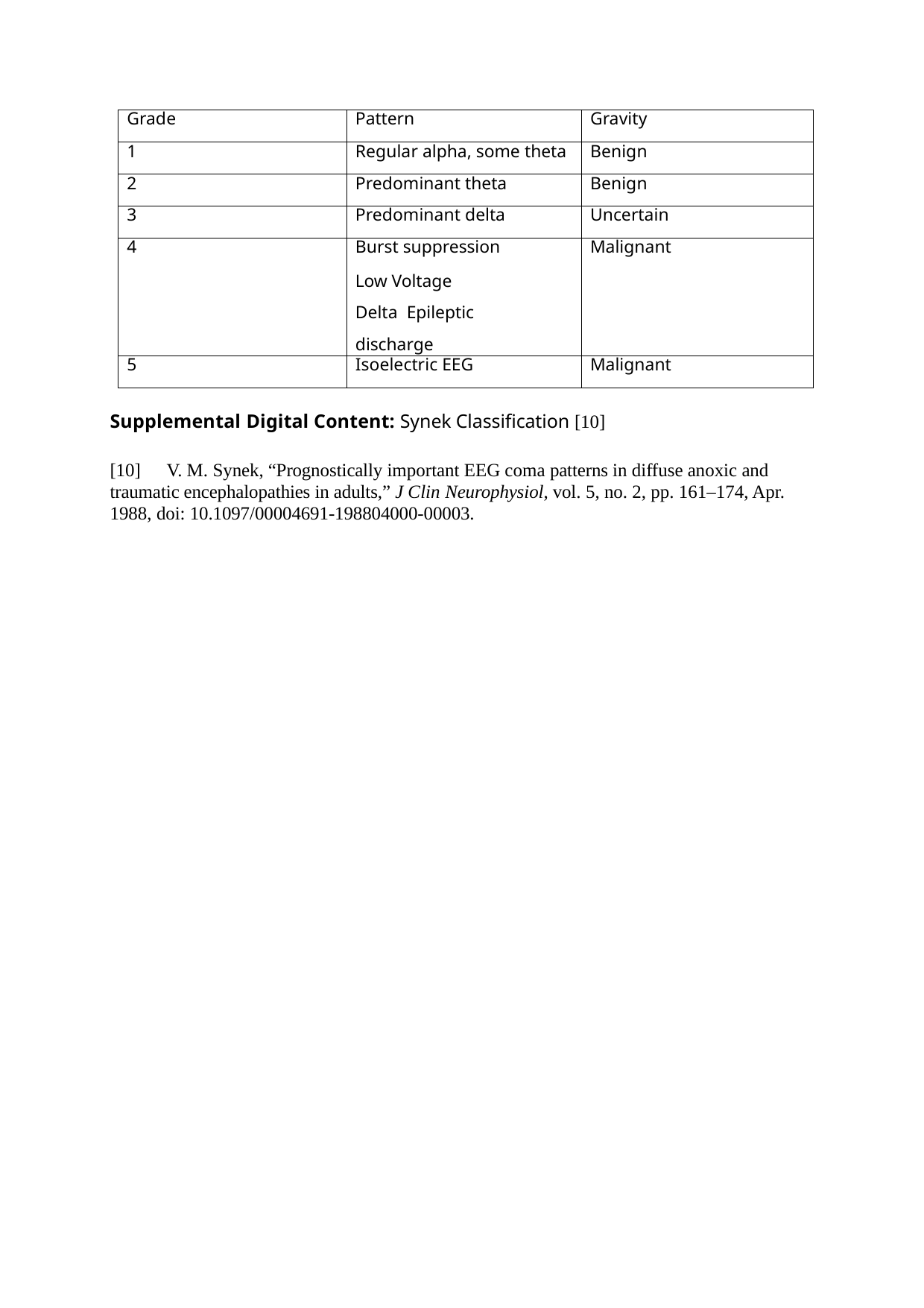

| Grade | Pattern | Gravity |
| --- | --- | --- |
| 1 | Regular alpha, some theta | Benign |
| 2 | Predominant theta | Benign |
| 3 | Predominant delta | Uncertain |
| 4 | Burst suppression Low Voltage Delta Epileptic discharge | Malignant |
| 5 | Isoelectric EEG | Malignant |
Supplemental Digital Content: Synek Classification [10]
[10]	V. M. Synek, “Prognostically important EEG coma patterns in diffuse anoxic and traumatic encephalopathies in adults,” J Clin Neurophysiol, vol. 5, no. 2, pp. 161–174, Apr. 1988, doi: 10.1097/00004691-198804000-00003.
